# Severe Intermittent Affections

**Published:** 1831

**Authors:** 


### SEVERE INTERMITTENT AFFECTIONS.

M. GUALTIER DE CLAUBRY has published two cases in the Journal Hebdomadaire, which deserve a short analysis.

*Case* 1. Mademoiselle D——, aged 79 years, (she ought to have taken the title of Mrs. ten years previously,) of robust constitution, living an austere life, and affected for nine years past with caries in the left hand, became affected in April, 1829, with conjunctivitis, and afterwards, in May, with erythema, and an obstinate pustular eruption on the forehead, accompanied by violent hemicrania. On the 22d May, she experienced some irritation in her bowels, attended by a diarrhœa. After a day's intermission, she was seized on the night of the 24th with pain in the right hypochondrium, shivering, pallor of the face, &c. to which succeeded the diarrhœa as before. 25th. Free from diarrhœa. 26th. The diarrhœa returned in the night, with the above-mentioned precursory symptoms. These subsided, and recurred on alternate days or nights, puzzling the doctor most sadly, till at length he could no longer be blind to the periodical type of the disorder. He then administered twenty grains of the sulphate of quinine in 24 hours, and put an end to the complaint.

*Case* 2. INTERMITTENT SOPOROSE AFFECTION.

In the month of April, 1830, the same female, who had continued to enjoy excellent health, considering her great age, and went regularly to church at six o'clock every morning, was taken suddenly with a chilliness, malaise, head-ache, and giddiness, so that she fell down in a state of stupor. The respiration was laborious, and the pulse was scarcely perceptible. This state continued for four hours, and was removed by sinapisms and pediluvia, ending in a general perspiration, and complete remission of all the symptoms. The succeeding day was passed in apparent health; but the day after that presented a repetition of the soporose state, still more marked than in the first instance. The medical attendant now became aware of the periodical character of the complaint; but was afraid, thus early, to have recourse to the quinine. He therefore ordered sinapisms to the legs, and leeches behind the ears, together with a purgative lavement. As soon as the comatose symptoms gave way, the tonic was ordered, in the dose of 12 grains in the 24 hours. The comatose attack recurred at the same hour, on the second day, as before, but much milder in force. The quinine was continued; and no more accessions took place.

We shall not indulge our readers with a train of reflections which arose in the reporter's mind, in consequence of the foregoing case. The results are so decided, and the conclusions so obvious, that it will be unnecessary to say any thing on this occasion.

---

